# Research on comprehensive evaluation & development of aesthetic education based on PCA and CEM model

**DOI:** 10.1371/journal.pone.0308446

**Published:** 2024-08-09

**Authors:** Xin-Hong Xu, Yu-Ting Niu, Zhi-Min Li, Yue-Yang Xu, Ke-Wang Cao

**Affiliations:** 1 School of Art, Anhui University of Finance and Economics, Bengbu, China; 2 Graduate School, Hankuk University of Foreign Studies, Seoul, Korea; 3 School of Statistics and Applied Mathematics, Anhui University of Finance and Economics, Bengbu, China; Far Eastern University - Manila, PHILIPPINES

## Abstract

Aesthetic education, conveyed through public art courses, serves as a vital form of humanistic literacy education. It represents an effective approach to fostering innovative and creative thinking among college students. In order to effectively analyze the aesthetic education work of 46 universities, an aesthetic education index evaluation system is constructed, involving indicators including faculty strength, curriculum setting, teaching management, artistic practice, and teaching support. The secondary indicators corresponding to the five indicators are statistically analyzed, and a comprehensive evaluation analysis of the current development status of aesthetic education in 46 universities in Anhui Province is conducted by combining theoretical analysis with empirical analysis. Based on principal component analysis, an integrated evaluation model for the development of aesthetic education in universities in Anhui Province is further constructed. The model designed quantifies the influence weight of each aesthetic education index on the development of aesthetic education in Anhui Province, and forges a theoretical basis for determining the precursors of rapid development of aesthetic education in Anhui Province. Additionally, a novel approach is introduced to gauge the progression of aesthetic education within universities in Anhui Province, considering the dispersion of aesthetic education index data across the province. The comprehensive evaluation model for the development of aesthetic education in Anhui Province exhibits an overall declining trend. Hence, it is suggested to utilize the maximum value of the first derivative of the comprehensive evaluation model as an indicator of the imminent rapid development of aesthetic education in Anhui Province. On this basis, the probability equation of sustainable development of aesthetic education in Anhui Province is defined. Overall, the research results lay a theoretical foundation for the development of aesthetic education in Anhui Province.

## 1. Introduction

Aesthetic education, also referred to as aesthetic or art education, holds significant importance within higher education. It typically pertains to undergraduate programs lasting four or five years, excluding vocational and technical colleges, as well as graduate education for the time being. Based on the *National General Art Education Curriculum Guidelines 2006 and the Opinions on Solidly Improving the Aesthetic Education Work in Colleges and Universities in the New Era 2020* [1, 2], the results of aesthetic education in colleges and universities over the past 15 years indicate that on the one hand, aesthetic education in colleges and universities is still in the stage of theoretical exploration and practical education, while on the other, there is no feasible evaluation system available for the actual effect of aesthetic education. This results in an imbalance between excessive aesthetic education theory and the lack of evaluation system.

According to relevant documents such as *China’s National Medium-to-Long-Term Education Reform and Development Plan 2010–2020* [3], *Opinions on Comprehensively Strengthening and Improving Art Education in Schools* [4], and *Regulations on Art Education Work in Schools* [5], this article examines 46 undergraduate universities in Anhui Province, adhering to principles of scientificity, comprehensiveness, and hierarchy. Meanwhile, it employs reverse reasoning and considers multiple observation points such as faculty strength, curriculum design, teaching management, artistic practice, and teaching support. Through the utilization of methods like principal component analysis, entropy weight method, and BP neural network method, the article offers a comprehensive evaluation of the current state of aesthetic education in universities in Anhui Province. Additionally, it highlights future challenges that need to be addressed.

## 2. Literature review

Aesthetic education is an education that cultivates people’s aesthetic perception, appreciation, creativity, and judgment. It holds irreplaceable significance in the higher education system, and presents long-term effectiveness, strong infectiousness, and transcends time, space, and ethnic and national aesthetic universality. Plato advocated for the cultivation of citizens in both body and mind as the ultimate goal of education, while Confucius emphasized the importance of poetry and music education as primary avenues for aesthetic education. Indeed, aesthetic education is an emotional education that concerns people mentality and personality development. When individuals are in crisis and pressure, aesthetic education can furnish them with a balanced, stable, and positive attitude to life [6]. Filipović and Vojvodić [7] believed that aesthetic education provides opportunities for students’ creative ability, creative communication ability, and aesthetic ability development, thereby affecting their personality emancipation and all-round development. In this way, art education makes people the most humanized and complete individual [8]. The implementation of aesthetic education in universities primarily involves utilizing public art courses as the foundation of art education. It centers on art works within the context of art history as the core content of the educational process. Art education refers to the education of music, dance, drama and visual arts disciplines [9]. Specifically, art encompasses various forms, including visual arts such as painting, drawing, sculpture, filmmaking, architecture, photography, and ceramic art. Additionally, it includes literary arts such as poetry, drama, prose, and novels, as well as performing arts like drama, dance, and music [10]. According to the *National Common Propaganda on Public Art Education in Colleges and Universities in China*, public art courses refer to those emphasizing aesthetics, art history and theory, art appreciation, and artistic practice represented by music, dance, painting, calligraphy, drama, film and television.

From the perspective of function and method of aesthetics, reconstructing STEM education by integrating art education is a way to implant art education [11]. STEAM combines science, technology, engineering, art, and mathematics education [12]. Meanwhile, art education can enhance students’ innovation ability and promote the cultivation of comprehensive innovative talents [13]. Scientific work requires creativity and critical thinking, while art and science mutually inspire each other in a subtle and covert way [14]. Wang and Zeng [15] believed that imaginative thinking ability is a kind of creative thinking ability, which is not only the core of art but also the key to scientific research. However, it is a challenge to scientifically evaluate the actual function of art in STEAM education from the student-centered perspective [16]. Teachers should have good adaptive teaching abilities to ensure that the aesthetic effect of public art courses is achieved [17]. In order to shift from the traditional aesthetic evaluation model centered on teachers, classrooms, and textbooks, a more student-centered approach can be adopted. This involves conducting aesthetic ability evaluations based on indicators such as artistic cultural knowledge and skills, artistic abilities, and artistic achievements. However, despite these efforts, the evaluation may still exhibit some bias towards a student-centered perspective [18]. In 2018, in order to construct a comprehensive evaluation system for higher education aesthetic education, Shandong Province established an evaluation index system for higher education aesthetic education work from five dimensions of curriculum design, teaching management, teacher team, artistic practice, and condition guarantee [19]. Only by adopting a more student-centered approach to aesthetic education evaluation can the aesthetic development of universities in the province truly benefit.

## 3. Comprehensive evaluation analysis

### 3.1 Construction of the comprehensive evaluation index

Aesthetic education as an educational phenomenon can be described, measured, calculated, and analyzed to help depict the quality of aesthetic education. Observing, describing, or researching the development of aesthetic education in various universities in Anhui Province aids in cultivating a reasoned comprehension of its status, distinctive features, and operational mechanisms. Comprehensive evaluation serves as a quantitative empirical method enabling the comparison of research objects through evaluation outcomes. Evaluating and comparing the progress of aesthetic education in universities across Anhui Province necessitates the establishment of a viable comprehensive evaluation index system, which should adhere to the following four principles:

The first principle is systematicity, where various indicators have logical relationships, reflecting the main characteristics and status of the overall system of aesthetic education from different aspects. The second is the principle of typicality, which emphasizes that indicators should possess certain representative characteristics to comprehensively reflect the current development status of aesthetic education. Selected indicators should have the capacity to guide the evaluation process effectively. The third principle involves independence, where the interaction of indicators within the system manifests as the characteristics of subsystems. The evaluation index system of aesthetic education includes independent subsystems reflecting teacher conditions, curriculum settings, teaching management, practical results, and teaching guarantees. The fourth principle encompasses comparability, operability, and quantifiability. The index system utilizes existing data and standards, ensuring consistency in the calculation methods and measurement of indicators. The indicators should be as concise as possible, with strong micro-level characteristics, easy to collect, and quantifiable.

Aesthetic education quality evaluation entails a comprehensive assessment and acknowledgment of the situation, processes, methods, objectives, and outcomes of education. This contributes significantly to the management, supervision, and enhancement of the quality and standard of education [20]. The evaluation of aesthetic education involves complexity and multidimensionality. A single indicator is far from sufficient to comprehensively and typically reflect its characteristics. Therefore, constructing a comprehensive evaluation index system requires decomposition, summarization, and refinement from multiple attributes, perspectives, and features [21]. Drawing on normative documents and prior scholarly research, an evaluation index system for the advancement of aesthetic education in universities within Anhui Province is hereby formulated, which comprises five sub-dimensions, i.e., teacher qualifications, curriculum design, teaching management, artistic practice, and teaching support, as delineated in Table 1.

**Table 1:** The development index system of aesthetic education in colleges and universities in Anhui Province.

	Primary index	Secondary index	Symbol
*Development of aesthetic education in colleges and universities in Anhui Province*	*Faculty*	Number of full-time teachers compared with actual teachers of public art courses	*A1*
		Number of aesthetic education papers published by teachers	*A2*
		Number of prizes awarded to outstanding instructors in Class B and above discipline competitions	*A3*
		Whether to organize skills training for art teachers	*A4*
		Whether to introduce art academy experts, folk art and intangible cultural traditions	*A5*
	*Curriculum provision*	Whether to open public art education elective courses	*B1*
		Whether to set up school-based aesthetic education course network resources or build network platform	*B2*
		Whether to require students to take at least one art limited course during school and pass the assessment	*B3*
		Number of public elective courses offered offline, such as calligraphy, Chinese folk art studies, paper-cutting or intangible cultural heritage projects	*B4*
	*Teaching management*	Number of specialized public art curriculum management departments and teaching institutions	*C1*
		Whether there are full-time staff in charge of aesthetic education institutions or teaching departments	*C2*
		Whether there are clear standards and requirements to publish the syllabus of public art courses	*C3*
		Whether to establish a special public art curriculum evaluation method and curriculum evaluation method	*C4*
		Whether to organize public art course evaluation, evaluation and teaching observation activities	*C5*
	*Artistic practice*	Number of traditional cultural associations accounting for the number of school-level art associations	*D1*
		Proportion of school-level and above art activities in the total number of activities	*D2*
		Number of art inheritance bases	*D3*
		Number of prizes won by students in Category B and above art competitions	*D4*
		Number of finalists in Anhui competition of National college students Art Exhibition	*D5*
		Cultural people into the campus/non-heritage into the campus/Hui Feng Wan Yun accounted for the proportion of aesthetic education funds	*D6*
		Whether there is art to help rural revitalization activities	*D7*
	*Teaching guarantee*	Total investment in aesthetic education	*E1*
		Number of art studios, theaters and studios	*E2*
		Number of art galleries and exhibition halls	*E3*
		Number of other fixed art venues such as showrooms, calligraphy copying rooms, graphic design rooms, printmaking design rooms and pottery rooms	*E4*

### 3.2 Data source and processing

To measure the development of aesthetic education in universities in Anhui Province, while ensuring the authenticity, validity, and accessibility of data, a variety of sources can be utilized. These may include annual reports on art development from each university, public documents from the Anhui Provincial Department of Education, official documents from each university’s website, and publications like the *China Education Statistical Yearbook*. These sources can be organized and compiled to form original cross-sectional data with a time variable set to 2020 for empirical analysis.

In a multi-indicator evaluation system, each indicator often varies in units and magnitudes due to their different nature. To address this, raw data undergo preprocessing, involving standardization and quantification of qualitative indicators. This ensures that all indicator values are on a uniform scale, facilitating comprehensive evaluation analysis.

For qualitative indicators, scoring can be completed by assigning "1" for "yes" and "0" for "no". Following that, a weighting factor judgment table method can be used to assign weights for quantification. The data standardization process in this paper mainly involves standardizing positive indicators, where max(*x*_*nm*_) and min(*x*_*nm*_) denote the maximum and minimum values of the nth indicator in the *m*_*th*_ year, *x*_*nm*,*k*_ refers to the original value of the nth indicator for school *k* in year *m*, and *z*_*nm*,*k*_ represents the standardized data. The calculation formula can be expressed as follows:

$$Z_{\text{nm},\text{k}}=\frac{{\text{x}}_{nm,k}-\text{min}(x_{nm})+0.00000001}{\text{max}(x_{nm})-\text{min}(x_{nm})}$$ (1)

This formula standardizes the raw data *x*_*nm*,*k*_ for each indicator across all schools and years, ensuring comparability across different indicators.

### 3.3 Method description

In comprehensive evaluations, utilizing different methods provides analyses from various perspectives. To overcome the many differences in preference and adaptability inherent in single comprehensive evaluation methods, employing multiple evaluation methods is essential, followed by the integration of results [22]. This paper takes the 46 universities in Anhui Province as samples, constructs an indicator system, and summarizes and analyzes the development of aesthetic education in each university. Methods such as the entropy method, BP neural network method, and principal component analysis method are employed, followed by a combined evaluation approach.

The entropy method explains the importance of indicators by the degree of variation in their values, serving as an objective and scientific method for determining weights [23]. The degree of variation in indicators is negatively correlated with information entropy and positively correlated with importance, with a smaller entropy of an indicator indicating a greater weight. Using the entropy method to determine the weights of various indicators in the evaluation can reasonably avoid the subjectivity caused by manual judgment. According to its calculation steps, m indicators are selected, with a total of n samples, where X_ij_ represents the value of the j_th_ indicator for the i_th_ sample, and the sample weight is first calculated:

$$P_{ij}=\frac{X_{ij}}{\sum_{i=1}^{n}X_{ij}}$$ (2)

Calculate the index entropy:

$$e_{j}=-K*\sum_{i=1}^{n}\left(P_{ij}*\text{ln}(P_{ij})\right),K=\frac{1}{\text{ln}\;n}$$ (3)

After calculating the coefficient of variation *d*_*j*_ = 1− *e*_*j*_, the essence of calculating the weights of evaluation indicators is to utilize the variation coefficient of the indicator information. The higher the variation coefficient, the greater the importance of the evaluation, and thus the larger the weight. This indicates a greater contribution to the evaluation results. The weight of the jth indicator is then calculated as follows:

$$w_{j}=\frac{d_{j}}{\sum_{j=1}^{m}d_{j}}$$ (4)

Finally, the comprehensive score of each sample can be expressed as follows:

$$z_{j}=\sum_{j=1}^{m}w_{j}x_{ij}$$ (5)

The Back Propagation (BP) neural network comprehensive evaluation method offers a comprehensive and interactive approach. It effectively bypasses the inaccuracies associated with manually assigning weights and the complexities of solving correlation coefficients. Moreover, it enables comprehensive evaluation of instances with large quantities and numerous indicators [24]. The BP neural network is an artificial neural network composed of input, hidden, and output layers. The input layer nodes only receive signal inputs, while the output layer nodes perform linear weighting, and the hidden layer nodes primarily handle the most significant mathematical processing of information. During forward propagation, training samples traverse through the input layer, followed by the hidden layer, and finally to the output layer. If the output results fail to meet the expected output, the process will enter the backpropagation stage. Herein, the weights and thresholds of each neuron are continuously adjusted based on the set prediction error until approaching the expected output. The aesthetic education development in Anhui Province’s universities is assessed by inputting indicators from teacher qualifications, curriculum design, teaching management, artistic practice, and teaching support subsystems into Python. Leveraging the potent nonlinear mapping capability of BP neural networks, the data undergo iterative learning and optimization for a comprehensive evaluation.

Principal Component Analysis (PCA) is a multivariate statistical analysis method that reduces the dimensionality of data by linear transformation to select a smaller number of important variables. The basic idea is to recombine the original set of correlated indicators into a smaller set of uncorrelated composite indicators *F*_*m*_ to replace the original indicators.


$$\left\{\begin{array}{l} {\text{F}}_{\text{1}}={\text{a}}_{11}X_{1}+a_{12}X_{2}+\cdots +a_{1p}X_{p}\\ {\text{F}}_{\text{2}}={\text{a}}_{21}X_{1}+a_{22}X_{2}+\cdots +a_{2p}X_{p}\\ \cdots \cdots \\ {\text{F}}_{\text{m}}={\text{a}}_{m1}X_{1}+a_{m2}X_{2}+\cdots +a_{mp}X_{p} \end{array}\right.$$ (6)

The above equation satisfies that F_i_ and F_j_ are uncorrelated, that is, cov(*F*_*i*_, *F*_*j*_) = 0, and have nonzero covariances. F_i_ is the linear combination of *X*_1_,*X*_2_,*X*_3_….*X*_*p*_ with the largest variance, that is, it is the combination of all linear combinations that are uncorrelated with each other and have the largest variance. It is constructed as the new variable indicator, namely the first, second,…, mth principal component of the original variable indicators. In practical applications, the specific steps of using Principal Component Analysis (PCA) are as follows: to standardize the original data; to calculate the covariance matrix; to find the eigenvalue *λ*_*i*_ of R and the corresponding eigenvector *a*_*i*_ of the orthogonalized unit; and to select principal components: through *m* in *F*_1_,*F*_2_,*F*_3_….. *F*_*m*_ is determined by the cumulative variance contribution rate *G*_*m*_.

Scores of aesthetic education in universities in Anhui Province are calculated, Principal Component Analysis is conducted using the Sklearn toolkit in Python, with the cumulative variance contribution rate set to be greater than 80%, and the scores for each university are finally computed.

### 3.4 Result interpretation

The entropy method, BP neural network and principal component analysis method are used to calculate the development scores of aesthetic education in 46 universities in Anhui Province. The weights of each index are shown in Table 2:

**Table 2:** Comprehensive evaluation index weight.

Index	*W1*	*W2*	*W3*
*A1*	0.0145	0.0644	0.0278
*A2*	0.0600	0.0551	0.0676
*A3*	0.1016	0.0636	0.0133
*A4*	0.0274	0.0281	0.0536
*A5*	0.0411	0.0410	0.0804
*B1*	0.0093	0.0137	0.0226
*B2*	0.0108	0.0231	0.0263
*B3*	0.0084	0.0281	0.0203
*B4*	0.0130	0.0336	0.0315
*C1*	0.0092	0.0203	0.0224
*C2*	0.0077	0.0153	0.0187
*C3*	0.0132	0.0341	0.0325
*C4*	0.0106	0.0254	0.0261
*C5*	0.0073	0.0141	0.0178
*D1*	0.0197	0.0717	0.0618
*D2*	0.0285	0.0677	0.0825
*D3*	0.2834	0.0549	0.0827
*D4*	0.0606	0.0546	0.0129
*D5*	0.0599	0.0491	0.0858
*D6*	0.0377	0.0827	0.0040
*D7*	0.0905	0.0446	0.0865
*E1*	0.0603	0.0507	0.0659
*E2*	0.0068	0.0224	0.0185
*E3*	0.0068	0.0160	0.0185
*E4*	0.0114	0.0256	0.0201

*W1, W2, and W3 represent the weights for the entropy method, The Back Propagation (BP) neural network method, and principal component analysis method, respectively.

The weights of the indicators calculated by the three methods vary. The high rankings of both the number of teaching and research papers published by teachers and the number of art inheritance bases underscore their significance in supporting the development of aesthetic education. Research papers serve as vital data supporting the advancement of aesthetic education, while art inheritance bases enable schools, associations, and enterprises to leverage their strengths. They facilitate the discovery and cultivation of aesthetic education models and help standardize and guide aesthetic education behavior effectively. Hence, it is not difficult to understand that these two indicators occupy a large weight. In addition, significant weight is placed on the number of teachers participating in discipline competitions rated B or above, the ratio of traditional cultural associations to school associations, investment in aesthetic education funds, and the number of awards received in the previous National College Students Art Performance in Anhui Province.

**Fig 1:**
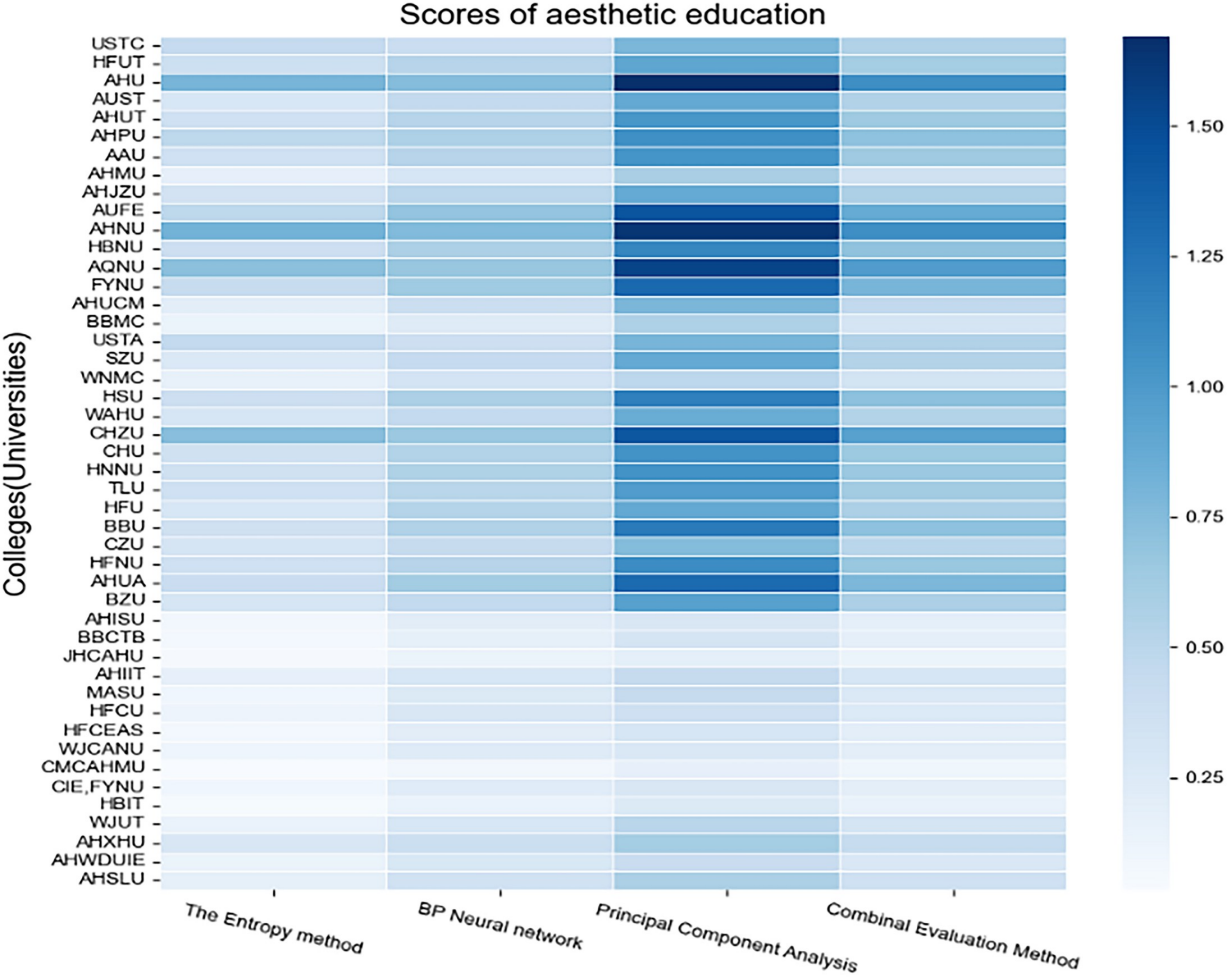
The score chart of the comprehensive evaluation.

After calculating the weights, the development scores of aesthetic education in 46 universities in Anhui Province are calculated according to three methods and evaluated jointly. Python software is used to draw the heat map, and the results are presented in visual form. The results are shown in Fig 1.

Fig 1 obviously reveals that darker colors correspond to higher scores, indicating a higher ranking in the development of aesthetic education. Observation over the last column of color blocks indicates that following the comprehensive evaluation, Anhui University, Anhui Normal University, Anqing Normal University, Chuzhou University, Anhui University of Finance and Economics, Fuyang Normal University, Anhui Academy of Fine Arts, Huangshan University, Anhui University of Engineering, and Bengbu University rank among the top ten. Reasons behind this include the fact that these universities offer five or more arts-related majors. Among them, Anhui Normal University, Anhui University, and Anqing Normal University offering over ten such majors. With sufficient funding and effective support for artistic venues, these universities prioritize professional faculty, thereby fostering comprehensive development in art courses, artistic heritage sites, and practical art endeavors. Additionally, institutions like Anhui Academy of Fine Arts specialize in cultivating artistic talents, positioning these universities at the forefront of aesthetic education development in the province.

## 4. Further analysis

**Fig 2:**
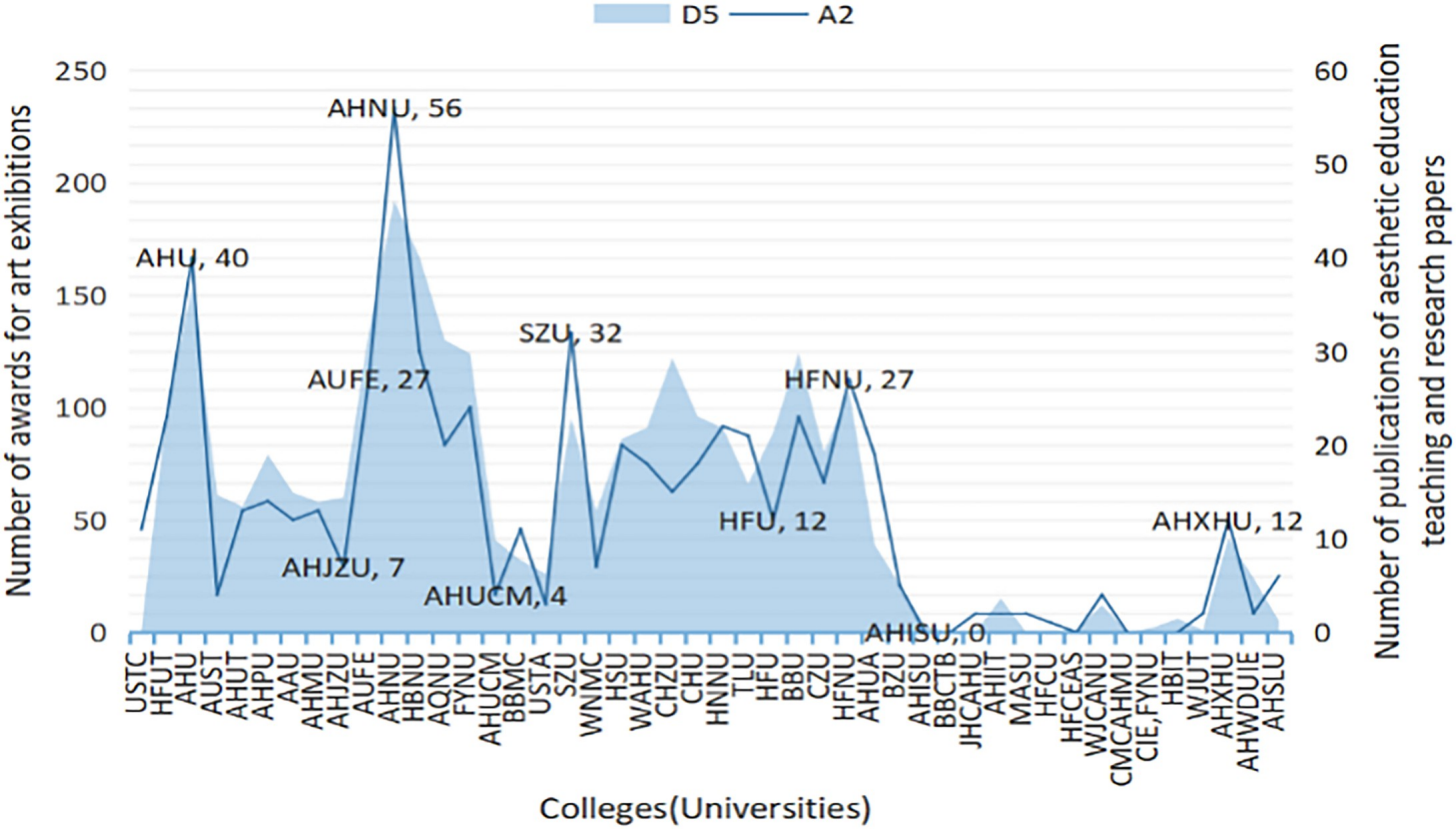
The number of teaching and research papers published in various universities and the number of awards in art performance.

Fig 2 illustrates a comparison of weighted indicators between research paper publications and the number of awards for artistic exhibitions across 46 universities in Anhui Province. From 2005 to 2022, in terms of the total number of awards at the national university art exhibitions organized by the Ministry of Education, universities with art schools, fine arts colleges, design colleges, and music colleges ranked prominently. A strong correlation between the quantity of research paper publications and the number of awards for artistic exhibitions is suggested by a calculated correlation coefficient of 0.899. Anhui Normal University, Anhui University, Suzhou University, Hefei Normal University, Anhui University of Finance and Economics, along with several other universities, exhibit a notable trend of both higher quantities of research paper publications related to aesthetic education and a greater number of awards for artistic exhibitions. This pattern implies the establishment of a systematic mechanism for nurturing talents, encompassing aspects such as faculty allocation, curriculum design, practical teaching methods, artistic practice, and participation in artistic exhibitions.

In terms of overall statistical award results, participation in artistic exhibitions and subject competitions serves as a dual-purpose platform. It not only allows universities to showcase their achievements in aesthetic education but also serves as an observation point for assessing the degree to which universities prioritize efforts in aesthetic education. Through retrospective analysis, with a focus on professional settings, faculty allocation, and curriculum design for aesthetic education, it becomes evident that certain universities excel in constructing flagship or advantageous disciplines. However, this specialization may lead to fewer entries in exhibitions and competitions, consequently resulting in fewer awards. This situation could potentially relegate aesthetic education to a subsidiary or even marginal position within these universities.

**Fig 3:**
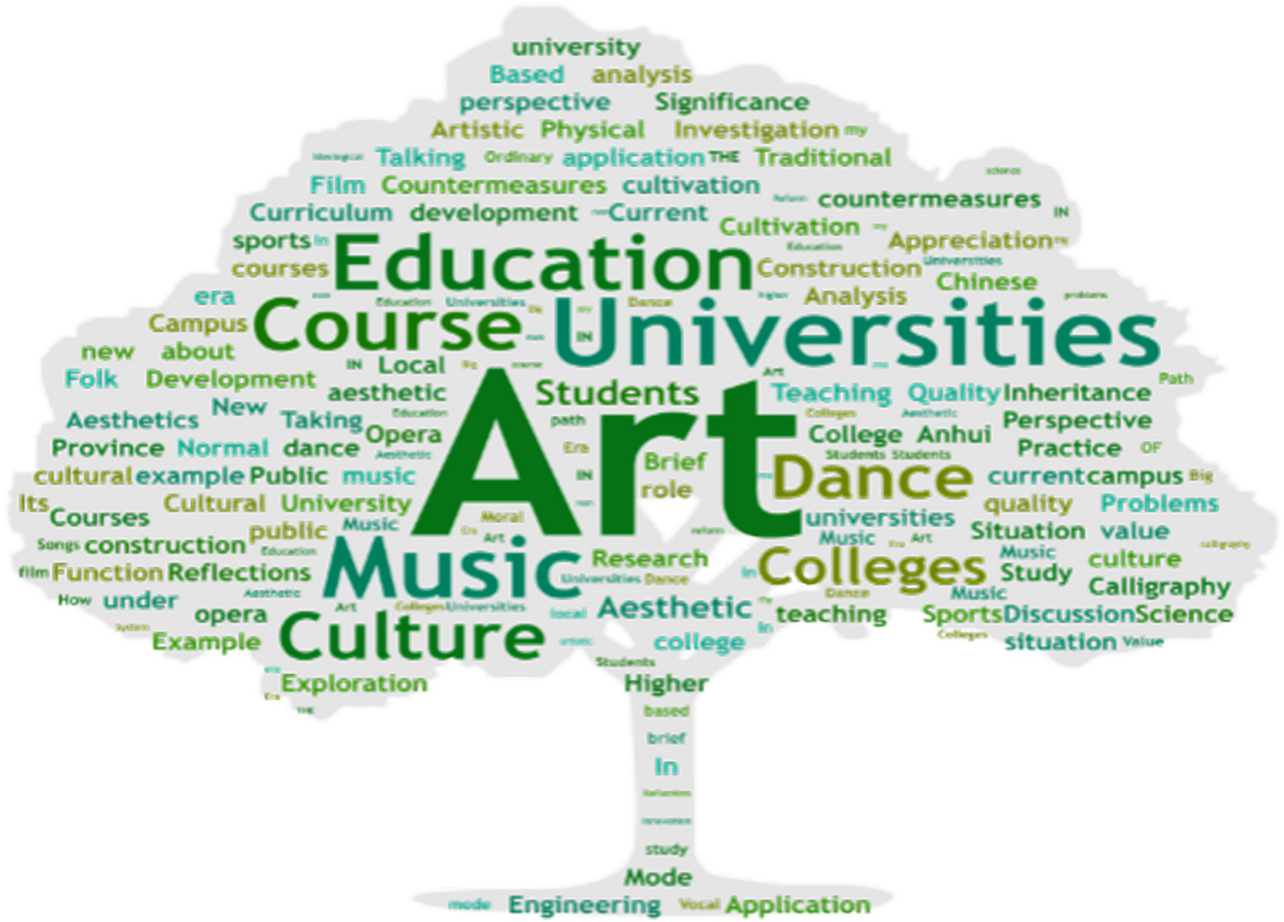
Word cloud of research papers.

An analysis is conducted on research papers regarding aesthetic education with significant weightage. Based on statistics from the China National Knowledge Infrastructure (CNKI) from 1990 to 2020, 624 research papers are examined. Python is employed for text segmentation and word frequency analysis to generate a word cloud, as shown in Fig 3. The word cloud demonstrates "aesthetic education," "curriculum," and "art" as the three most frequently occurring terms, suggesting that art education, primarily focusing on public art courses, holds a dominant position. Among these, music and dance often occupy the forefront of aesthetic education. However, courses such as art appreciation, film and television appreciation, drama appreciation, calligraphy appreciation, and traditional Chinese opera appreciation are relatively marginalized. This is attributed to the prevalence of music and dance education at the primary and secondary school levels. Statistics also reveal that 26% of universities do not offer courses related to calligraphy, Chinese folk art, paper cutting, or intangible cultural heritage. Notably, Chinese calligraphy education appears to lack prominence, accounting for only 0.048% of research paper contributions. Furthermore, through a parallel comparison of public art courses, the structural imbalance in course offerings highlights the uneven distribution of such courses among the 46 universities.

**Fig 4:**
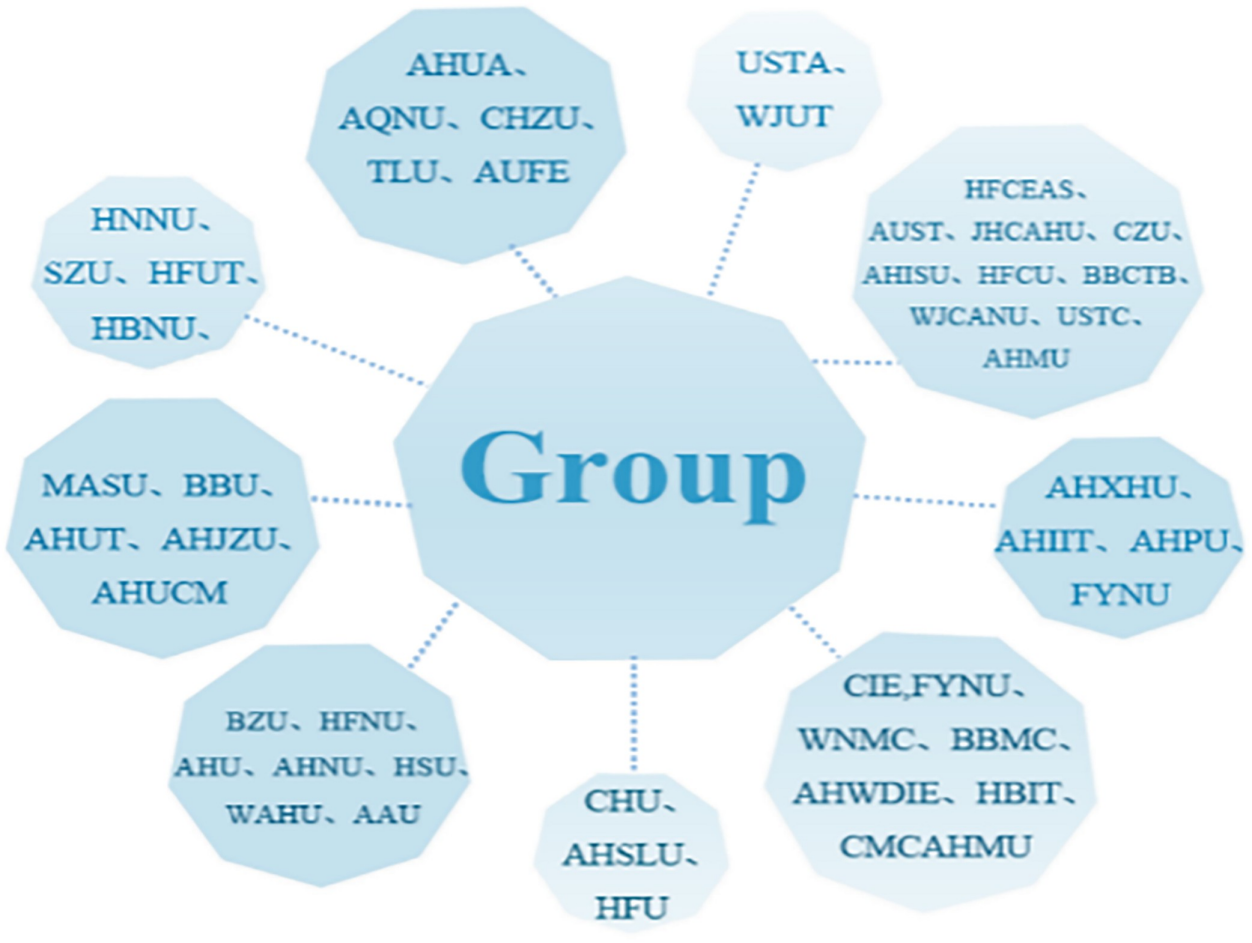
Display of cluster analysis results.

Differences in aesthetic education development in teaching management among the 46 universities in Anhui Province are further explored. Considering the small sample size and the teaching management indicators being categorical variables, a systematic cluster analysis method is adopted. The Ward method is used to calculate the distances between clusters using the squared Euclidean distance, and the number of clusters is determined based on the constructed pseudo-F statistic and pseudo-t^2^statistic. According to the statistics in Table 3, nine clusters are finally obtained, as shown in Fig 4.

**Table 3:** The pseudo-F statistics and pseudo-t2 statistics.

Number of clusters	Calinski/Harabasz pseudo-F	Duda/Hart	
		Je(2)/Je(1)	pseudo T-squared
2	18.71	0.4648	19.57
3	18.01	0.6742	12.08
4	20.03	0.3519	31.32
5	25.68	0.0000	.
6	29.01	0.2308	23.33
7	33.26	0.5185	5.57
8	38.01	0.4550	5.99
9	47.88	0.2857	5.00
10	64.43	0.0000	.
11	85.11	0.0000	.

The aesthetic education evaluation index system encompasses both basic statistics and variables. Hard indicators in teaching management include the presence of specialized public art course management departments, the number of teaching institutions, and the count of full-time staff in aesthetic education institutions or teaching departments. They have specific data requirements and are highly operational. The aesthetic management departments of the 46 universities vary widely, involving departments such as Art Education Centers, Cultural Arts and Quality Education Departments, Public Art Education Centers, Aesthetic Teaching Departments, Undergraduate Art Research Offices, Undergraduate Art Education Centers, Sports and Art Clubs, Public Art Education Clubs, Aesthetic Research Offices, etc. The diversity of management departments implies the flexibility of aesthetic education work, indicating an exploratory and practical stage in aesthetic education at universities. First, professional faculty and aesthetic education faculty may share resources, but this arrangement is uncertain and does not form a fixed teaching team. At the same time, soft indicators include the teaching syllabus of public art courses, the methods of public art course evaluation and assessment, public art course evaluations, awards, and teaching observation activities. Among these, cultural courses offered by engineering and medical universities occupy a relatively high proportion, but they are somewhat related to aesthetic education. The absence of standardized indicators among provincial universities limits the effectiveness of assessing procedural soft indicators, making it challenging to operationalize. The convergence of hard and soft indicators leads to a diverse range of teaching management approaches.

As shown in Fig 4, a cluster analysis is conducted on the 46 universities in Anhui Province based on teaching management. The results indicate that Anqing Normal University, Chuzhou University, Anhui University of Finance and Economics, Tongling University, and Anhui Academy of Fine Arts are grouped into one cluster; Anhui University, Anhui Normal University, Anhui Agricultural University, Huangshan University, Bozhou University, Anhui Science and Technology University, and Hefei Normal University form another cluster; Bengbu Medical College, Wannan Medical College, Anhui Wenyi Information Engineering College, Fuyang Normal University School of Information Engineering, Anhui Medical University Clinical Medicine College, and Huaibei Normal University constitute a third cluster. The distribution of different types can be attributed to the setup of teaching management institutions. Specifically, 10 universities have dedicated institutions solely responsible for aesthetic education, supported by full-time staff. In contrast, 36 universities lack such dedicated staff, resulting in the delegation of aesthetic education responsibilities to various management departments. Regarding the establishment of art-related majors, six universities have more than 10 art-related majors, eight have between 7 and 9 art-related majors, 13 have between 4 and 6 art-related majors, 10 have between 1 and 3 art-related majors, and eight do not have any art-related majors. In terms of the student-teacher ratio for aesthetic education, according to the *Guidelines for Public Art Courses in National Regular Higher Education Institutions*, the number of teachers responsible for teaching public art courses in each school should account for 0.15% to 0.2% of the total number of students, with full-time teachers taking up 50% of the total number of art teachers. Based on the given ratio, certain medical and science/engineering universities have only one-third the necessary number of instructors for public art classes. Furthermore, universities with adequate faculty often fail to differentiate between art educators dedicated to specialized training and those teaching public art courses, thereby not offering a comprehensive array of public art courses. In terms of teaching management, responsibilities and personnel regarding faculty, courses, and practical activities should be further refined.

## 5. Aesthetic education comprehensive evaluation model

### 5.1 Establishment of the model

Herein, multiple indicators of aesthetic education development are selected to analyze and evaluate the status of aesthetic education in universities in Anhui Province. However, it remains challenging to quantitatively analyze aesthetic education development. The difficulty arises partly from some indicators being binary outcomes and the potential for the research conclusions to be limited to specific scenarios if only intuitive analysis of aesthetic education indicators is conducted. Therefore, five nonlinear indicators are hereby selected, which include the number of teacher publications on aesthetic education, the frequency of organizing school-level and above art activities, the number of awards in Anhui University Student Art Exhibition, the number of awards in provincial-level arts competitions, and the proportion of funding for cultural and folk heritage activities on campus-as the benchmark indicators for principal component analysis. A comprehensive evaluation model for aesthetic education development, considering data dispersion, is established. If the original variables are denoted as *x*_1_,*x*_2_,⋅⋅⋅⋅,*x*_*n*_, and the new variables after principal component analysis are denoted as *F*_1_,*F*_2_,⋅⋅⋅⋅,*F*_*m*_, where m < n, the new variables are linear combinations of the original variables. The new variable coordinate system is derived by translating and rotating the original one. The resulting space, comprising the new variables, is termed the m-dimensional principal hyperplane. On this plane, the first principal component F_1_ corresponds to the direction of the largest data variation (contribution rate e1), while for F, there are *e*_2_ ≥ *e*_3_ ≥ ⋅⋅⋅≥ *e*_*m*_ in sequence. As a result, the new variables effectively encapsulate the essence of the original data, while the m-dimensional hyperplane holds the utmost information content. Despite the possible presence of a slight loss of data information, this method effectively captures the primary contradictions, extracting the majority of variance from the original variables. By reducing the number of variables and focusing on the essential information, it facilitates problem analysis and processing, streamlining the overall approach.

**Fig 5:**
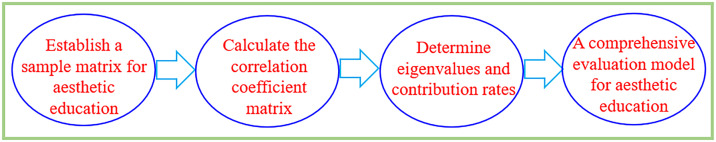
Calculation process of the comprehensive evaluation model.

Through principal component analysis, some of the original data information with minor contributions to the final results can be eliminated, while the main characteristic information of aesthetic education development indicators is retained. This is conducive to the analysis and processing of aesthetic education development in Anhui Province. The calculation process of the comprehensive evaluation model for aesthetic education is illustrated in Fig 5.

To standardize the spacing between different information parameters and to represent the authenticity of the original data as finely as possible, this study employs the investment funds allocated to aesthetic education in universities in Anhui Province as the independent variable. The matrix is composed of column vectors representing aesthetic education evaluation indicators. Thus, the sample matrix for aesthetic education in Anhui province can be obtained as follows:

$$F=(X_{1},X_{2},X_{3},X_{4},X_{5})=\left[\begin{array}{ccccc} 0.00835 & 0.110 & 0.0144 & 0.325 & 1.000\\ 0.02110 & 0.111 & 0.0135 & 0.321 & 0.929\\ \vdots & \vdots & \vdots & \vdots & \vdots \\ 1.000 & 1.000 & 1.000 & 1.000 & 0 \end{array}\right]$$ (7)

where, *F* denotes a 100×4 two-dimensional matrix, while *X*_1_,*X*_2_,*X*_3_,*X*_4_ and *X*_5_ respectively represent column vectors with varying steps for the number of papers published by teachers in aesthetic education, the number of school-level and above art activities conducted, the number of awards won in the Anhui division of college student art exhibitions, the number of awards won in provincial-level B-category and above art competitions, and the proportion of funds allocated for cultural heritage projects entering campuses.

Meanwhile, normalization processing is performed on five non-linear indicators of aesthetic education development, namely, the number of papers published by teachers in aesthetic education, the number of school-level and above art activities conducted, the number of awards won in the Anhui division of college student art exhibitions, the number of awards won in provincial-level B-category and above art competitions, and the proportion of funds allocated for cultural heritage projects entering campuses. This process yields a two-dimensional matrix representing aesthetic education development. Based on this two-dimensional matrix, the correlation coefficient matrix for aesthetic education development in Anhui Province can be calculated as follows:

$$\eta =\left[\begin{array}{ccccc} 1.0000 & 0.9854 & 0.8693 & 0.9935 & -0.9690\\ 0.9854 & 1.0000 & 0.9103 & 0.9596 & -0.9174\\ 0.8693 & 0.9103 & 1.0000 & 0.8247 & -0.8056\\ 0.9935 & 0.9596 & 0.8247 & 1.0000 & -0.9863\\ -0.9690 & -0.9174 & -0.8056 & -0.9863 & 1.0000 \end{array}\right]$$ (8)

The eigenvalues can be calculated based on the feature matrix using the following formula:

$$\left|\eta \text{-}\left.\lambda_{i}E\right|\right.=0$$ (9)

where, *λ*_*i*_ represents the eigenvalues of the correlation matrix, with i being 1, 2, 3, 4, 5; and *E* refers to the identity matrix.

The magnitude of eigenvalues in the correlation matrix directly reflects the amount of information they contain regarding Anhui Province’s art education data. Optimal selection of principal components relies on these eigenvalues, aiming to minimize their count. Traditionally, this entails choosing principal components where eigenvalues surpass 1 or ensuring that the cumulative contribution rate of eigenvalues is no less than 85%. The expression is as follows [25–27]:

$$\frac{\lambda_{i}}{\sum_{i=1}^{4}\lambda_{i}}\times 100\%\geq 85\%$$ (10)

Following the principal component analysis, the eigenvalues of the art education matrix in Anhui Province are calculated to be 0, 0.0019, 0.0552, 0.2497, and 4.6932, respectively. The corresponding eigenvectors can be obtained as follows:

$$u=(u_{1},u_{2},u_{3},u_{4},u_{5})=\left[\begin{array}{ccccc} 0.8256 & 0.1394 & 0.2583 & -0.1448 & -0.4595\\ -0.3513 & -0.4786 & 0.6492 & 0.1380 & -0.4550\\ 0.0004 & 0.1412 & -0.3637 & 0.8198 & -0.4192\\ -0.4392 & 0.6960 & 0.0289 & -0.3394 & -0.4547\\ 0.0449 & 0.4972 & 0.6153 & 0.4157 & 0.4465 \end{array}\right]$$ (11)

where, *u*_1_,*u*_2_,*u*_3_,*u*_4_ and *u*_5_ represent the eigenvectors corresponding to the eigenvalues 0, 0.0019, 0.0552, 0.2497, and 4.6932, respectively.

Based on the eigenvalues, one principal component is determined for the development of art education in Anhui Province. The numerical values of the associated eigenvectors, linked to the largest eigenvalue, act as the coefficients of the variables within the principal component function. These coefficients are commonly referred to as factor loadings of the principal component function. Consequently, the comprehensive evaluation model for the development of art education in Anhui Province is established as follows:

$$g=a_{1}x_{1}+a_{2}x_{2}+a_{3}x_{3}+a_{4}x_{4}+a_{5}x_{5}$$ (12)

where, g represents the comprehensive evaluation model; x_1_, x_2_, x_3_, x_4_ and x_5_ respectively denote the number of papers published by teachers on art education, the number of school-level and above artistic activities, the number of awards won by college students in Anhui competition area, the number of awards won in provincial-level B-class and above artistic competitions, and the proportion of funding for cultural heritage activities in schools; and a_1_, a_2_, a_3_, a_4_ and a_5_ refer to the weights corresponding to each indicator. Their values represent the ratio of each factor loading to the sum of the absolute values of all factor loadings. The expression is as follows:

$$a_{i}=\frac{h_{i}}{\left|h_{1}\right|+\left|h_{2}\right|+\left|h_{3}\right|+\left|h_{4}\right|+\left|h_{5}\right|}$$ (13)

where, *h*_i_ represents the factor loading, with i being 1, 2, 3, 4, 5.

According to Eq (13), the comprehensive evaluation model can be calculated as follows:

$$g=-0.206x_{1}-0.204x_{2}-0.188x_{3}-0.203x_{4}+0.199x_{5}$$ (14)

### 5.2 Precursors of rapid development in art education

The weight analysis in Principal Component Analysis (PCA) establishes a theoretical foundation for pinpointing sensitive indicators that mirror the advancement of art education in Anhui Province. Through PCA, a comprehensive evaluation model of art education development in Anhui Province is constructed, which incorporates multiple parameters such as the number of published papers on art education by teachers, the frequency of organizing arts events at the school and higher levels, the number of awards won by university students in the Anhui division of art exhibitions, the number of awards won in arts competitions at the provincial level and above, and the proportion of funding allocated to cultural heritage and folk art projects entering campuses. This model streamlines a comprehensive analysis of art education development in Anhui Province. Its essence lies in summing the product of each indicator and its corresponding weight across different dimensions of art education in higher education institutions within the province. A thorough investigation into the precursors of rapid development in art education aids in understanding the influence of various factors such as societal, policy, and technological factors on art education. It provides a basis for devising more effective strategies for art education development and lays the theoretical groundwork for the sustainable development of art education in Anhui Province.

**Fig 6:**
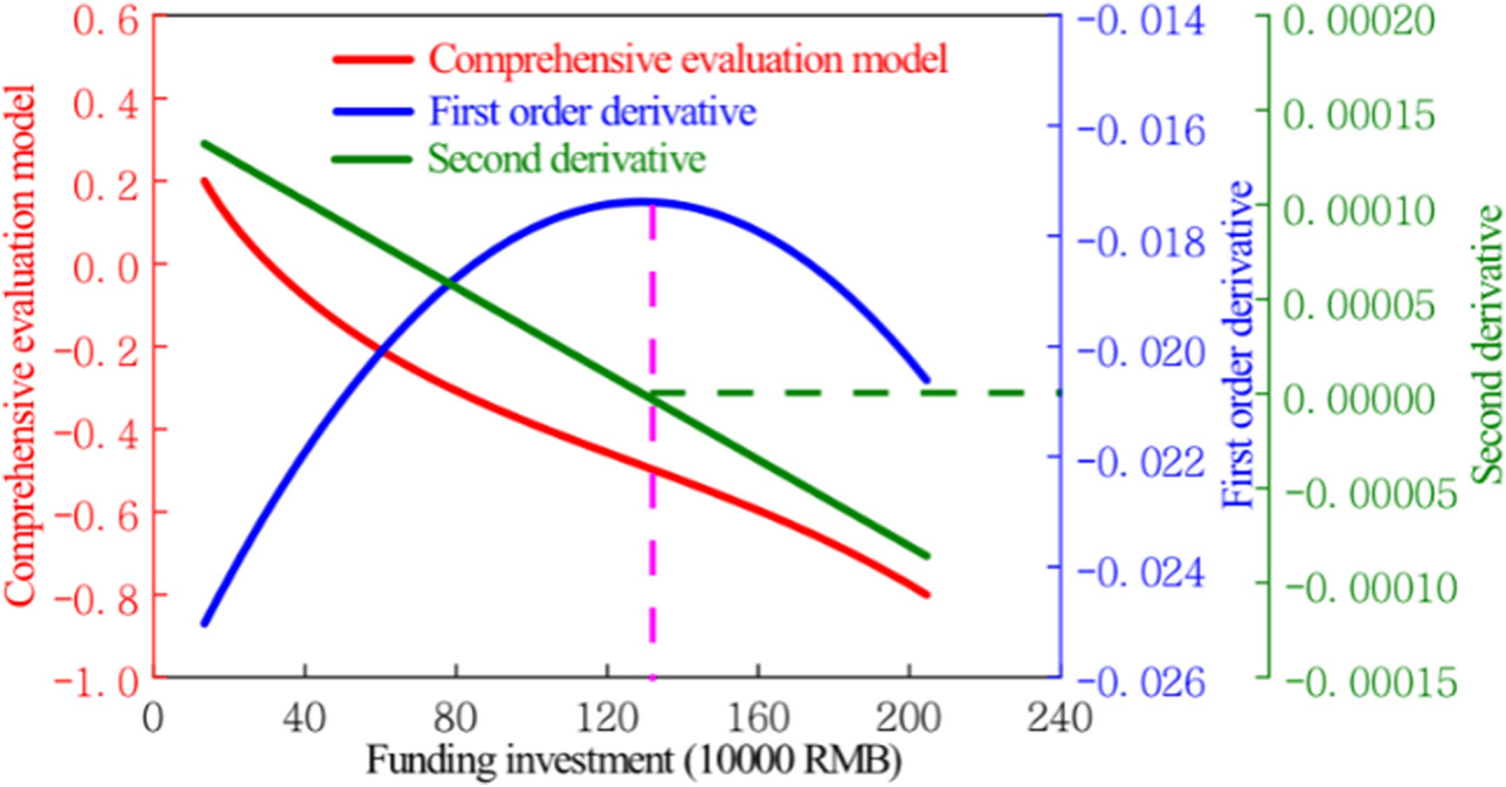
Evolution diagram of the comprehensive evaluation model of aesthetic education development in colleges and universities in Anhui Province.

Fig 6 depicts the evolution curve of the comprehensive evaluation model of art education development in Anhui Province as a function of funding input. As illustrated in the figure, the curve of the comprehensive evaluation model for art education development in Anhui Province exhibits an overall decreasing trend, with values ranging from -0.8 to 0.2. Despite the variations in numerical values and diverse changing trends among different art education indicators, indicating a certain degree of dispersion, this study takes into account the dispersion of data across these indicators. Polynomial fitting is applied to the curve of the comprehensive evaluation model for art education development in Anhui Province, using logarithmic, exponential, and polynomial functions. It is found that the correlation coefficient of the fitting curve reaches its highest value of 0.999 when a third-degree term is used, indicating a near-complete overlap between the fitting curve and the comprehensive evaluation model curve. Subsequently, the equation of the fitting curve is differentiated once and twice. The first derivative’s significance lies in indicating the rate of change of the curve in the comprehensive evaluation model for art education development in Anhui Province. It indirectly reflects the model’s sensitivity to changes in art education funding. Extreme values of the first derivative signify transitions in sensitivity states. The derivative curve of the comprehensive evaluation model for art education development in Anhui Province is shown in Fig 6, which exhibits an overall increasing followed by decreasing trend. As art education funding increases, the first derivative peaks, with the second derivative reaching 0. Herein, the authors pinpoint this maximum of the first derivative as the precursor to rapid development in art education. At this juncture, funding input for art education in Anhui Province stands at 1,331,000 RMB, presenting a corresponding comprehensive evaluation model value of -0.502.

### 5.3 Sustainable development probability of aesthetic education

Art education holds considerable significance in the sustainable development of society, emphasizing the cultivation of students’ creative thinking and innovation skills, both essential components for the enduring development of society. Creativity is a key factor driving social progress and problem-solving. Art education stimulates individuals’ independent thinking, fostering creativity and providing ongoing momentum for societal innovation. It helps to inherit and promote local culture while also facilitating communication and integration between different cultures. Art facilitates a deeper understanding and respect for multiculturalism, fostering a more harmonious and inclusive society. Besides, art education emphasizes cultivating students’ aesthetic tastes and cultural literacy, enriching individuals in the arts and enhancing their overall comprehensive qualities. Additionally, participation in artistic activities cultivates skills in interpersonal communication, cooperation, and collaboration, which are also crucial for societal sustainable development. The cultural and creative industries are integral components of modern society’s economy. Also, art education nurtures creative talents like artists, designers, and cultural professionals, fostering the development of the creative industry and establishing a cultural and economic foundation for societal sustainability. Moreover, it cultivates environmental awareness by drawing attention to environmental issues. At the same time, artworks serve as powerful tools for conveying environmental concepts, arousing concern through visual and aesthetic means, and advocating for sustainable environmental development.

The comprehensive evaluation model established in this paper serves as a quantitative analysis model for assessing the level of art education development. It takes into account the discreteness of art education indicator data and introduces a novel method for evaluating art education development. However, the comprehensive evaluation model cannot measure the characteristics of sustainable development in art education. Therefore, attempts are made to define the probability value of art education sustainable development based on the comprehensive evaluation model of art education in Anhui Province. From a superposition perspective, considering that the probability density function values for art education sustainable development must be non-negative, and given the observation from Fig 6 that the comprehensive evaluation model value of art education in Anhui Province is negative, it is not feasible to directly utilize the comprehensive evaluation model value as the probability density function value. According to the superposition method, the total energy expression based on the comprehensive evaluation model of art education is established as follows:

$$E(t)=\sum_{0}^{t}{\left(a_{1}x_{1}+a_{2}x_{2}+a_{3}x_{3}+a_{4}x_{4}+a_{5}x_{5}\right)}^{2}$$ (15)

where, *E*(*t*) represents the total energy of the comprehensive evaluation model for art education development; t stands for the funds invested in art education; and *x*_1_,*x*_2_,*x*_3_,*x*_4_ and *x*_5_ are parameters related to the investment in art education.

The total energy expression of the comprehensive evaluation model of Anhui Province’s art education is used as the probability density value, following the calculation formula of probability distribution functions in probability theory and mathematical statistics textbooks. Therefore, according to the superposition method [28], the probability of sustainable development of art education in Anhui Province can be expressed as follows:

$$P(t)=\frac{\int_{0}^{t}E(x)dx}{\int_{0}^{T}E(x)dx}$$ (16)

where, *P*(*t*) denotes the probability of sustainable development of art education in Anhui Province for any value of art education funding, and T refers to the peak value of art education funding.

**Fig 7:**
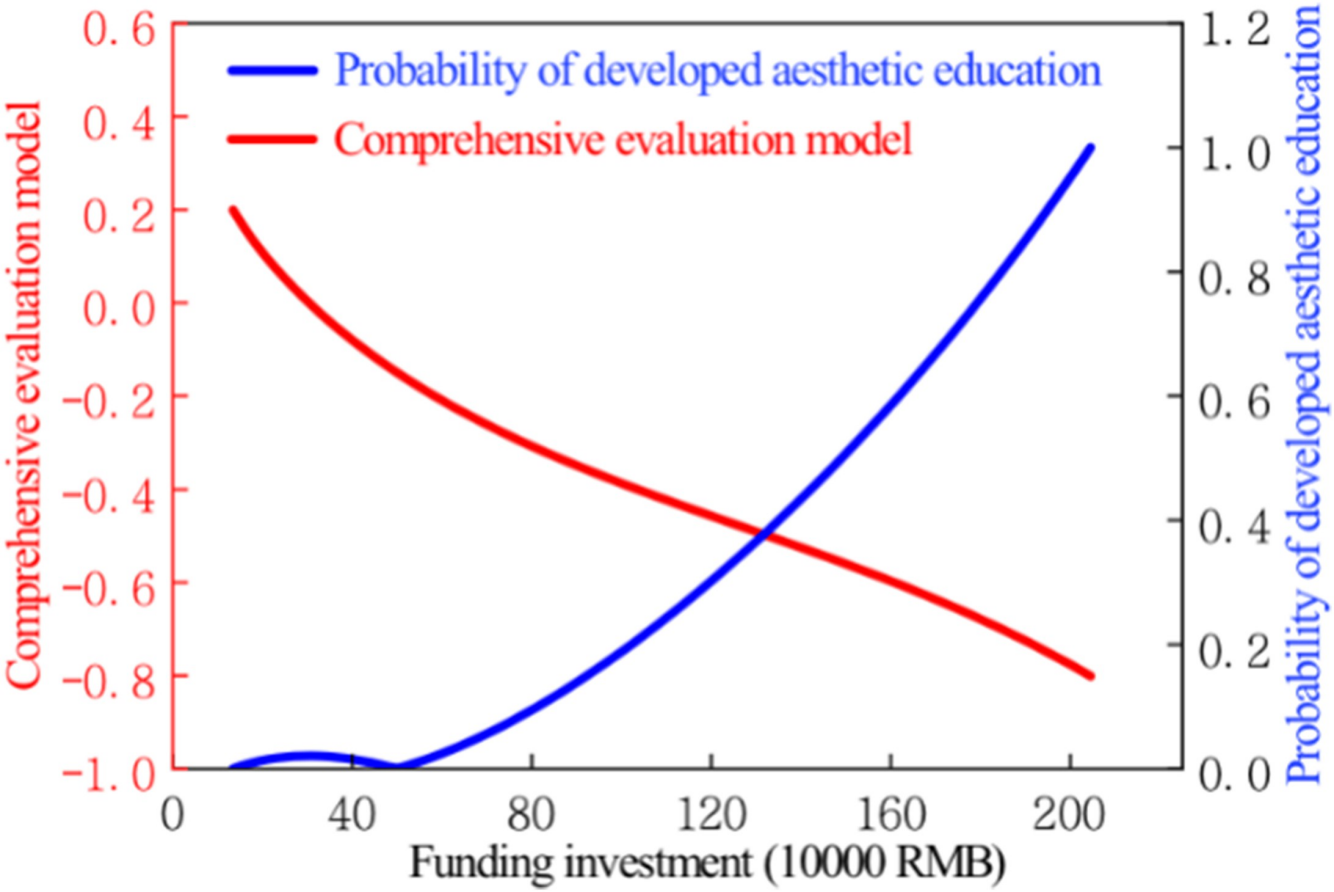
Evolutionary chart of the probability of sustainable development of art education in Anhui Province’s universities.

When t = T, the art education funding input in Anhui Province reaches its peak. Hence, the probability value of sustainable development of art education at the peak state is 1. By computing the probability density curve of sustainable development of art education in Anhui Province based on Eq (16), Fig 7 illustrates the evolutionary trend of the probability density of sustainable development of art education in Anhui Province. As shown in the figure, the probability density curve of sustainable development of art education in Anhui Province exhibits an overall trend of accelerating increase. The probability value of sustainable development corresponding to the precursor point of rapid development of art education in Anhui Province is 0.388. The probability values of sustainable development of art education corresponding to art education funding of 1.85 million yuan and 1.95 million yuan are 0.8 and 0.9, respectively. Examining the probability curve of sustainable art education development in Anhui Province provides access to probability values for sustainable development across different levels of art education funding.

## 6. Discussions and recommendations

In Anhui Province, 10 colleges and universities offer one to three art majors, while 8 lack any art programmes. Some medical and scientific institutions have a mere one-third of the public art faculty compared to the program’s needs. Additionally, colleges with adequate faculty do not segregate those focused on professional art education from public art teachers, resulting in an incomplete public art curriculum. The authors believe that a sound teaching management organisation should be established. On the basis of the 10 colleges and universities that already have had an organisation specifically responsible for aesthetic education, it is recommended that a specialised aesthetic education management organisation with full-time staff also be established in the remaining colleges and universities. This ensures a specialised team is dedicated to overseeing and advancing aesthetic education. Secondly, the setting of art majors should be optimised. For the 8 colleges and universities without art majors, more relevant majors should be gradually set up to enrich the disciplines. Meanwhile, institutions with a limited number of art majors should be motivated to expand their offerings and evolve towards a more diverse and holistic approach. This strategy aims to accommodate the varied demands of society for artistic talent and to bolster the overall competitiveness of these colleges and universities. Colleges and universities, particularly those focused on medical and technical fields, should bolster their recruitment efforts for public art programs. This is to ensure that the faculty count meets the necessary standards, thereby enhancing the quality and breadth of aesthetic education. In addition, it is recommended that full-time art teachers and public art course teachers be divided to undertake the teaching tasks of professional education and public art courses, respectively. This ensures that the courses are fully opened. In terms of teacher management, the teacher training and assessment mechanism should be strengthened, and art teachers should be organised to participate in training and exchange activities on a regular basis, thereby improving their teaching ability and professionalism. At the same time, a scientific teacher appraisal system can be established to evaluate the teaching effectiveness and performance, so as to motivate the teachers for consistent self-improvement and optimisation of the teaching quality. In terms of curriculum, the public art curriculum system should be enriched and improved. In alignment with the distinct profiles of colleges and universities and the varied interests of their students, a diverse curriculum of art courses, spanning music, visual arts, dance, theater, and other disciplines, should be established. This curriculum is designed to cater to the broad artistic learning needs of students, enrich their practical experience, and enhance their practical skills and overall quality through a combination of campus-based and extramural internships and art practice activities.

Finally, delineating clear responsibilities and assigning specific roles is essential. Create a personalized responsibility system to ensure that the task of aesthetic education work is decomposed into specific posts and individuals, so that every task has a responsible person in charge and is subject to proper supervision. By clearly defining roles and enforcing accountability, work efficiency is enhanced, ensuring the seamless execution of aesthetic education initiatives. For example, by adding a number of art majors and actively bringing in high-calibre teachers, Zhejiang University has rapidly formed a competitive cluster of art disciplines and increased the university’s influence in the field of art education. Meanwhile, the Chinese University of Hong Kong has significantly enhanced students’ artistic qualities and practical abilities through the provision of diversified art courses and the organisation of rich art practice activities. However, the above proposal also faces challenges on various fronts, including problems in funding, resources, time and management co-ordination. Specifically, the introduction of additional specialisations necessitates considerable financial and resource investment, potentially burdening HEIs with scarce resources. Recruiting and training adequate faculty, notably in medical and polytechnic institutions, is a protracted process. Crafting and launching a varied curriculum demands significant time and resources, potentially overwhelming current educational capacities. Moreover, organizing and overseeing practical sessions involves multi-party resource coordination, presenting logistical challenges. In order to achieve an overall improvement in aesthetic education, successful implementation of these recommendations requires detailed planning and step-by-step progress by the universities according to their own specific conditions, balancing short-term goals with long-term development.

The comprehensive evaluation model established in this paper serves as a novel method for quantitatively analysing the development degree of aesthetic education. Its theoretical basis relies on the discrete characteristics of aesthetic education metrics, utilizing mathematical curve-fitting techniques, along with the application of primary and secondary derivatives for analysis. The model can effectively analyse the relationship between the input of aesthetic education and the degree of development. The primary derivative reflects the sensitivity of the comprehensive evaluation model to changes in the funding of aesthetic education, while the point of extreme value indicates a shift in the state of sensitivity. The extreme value point of the primary derivative is considered the precursor point of rapid development of aesthetic education, similar to the inflection point in the theory of marginal effect, revealing the rapid growth of aesthetic education input funding upon reaching a certain degree. In order to measure the sustainable development characteristics of aesthetic education, the authors have integrated the concept of probability value. They have delineated the probability of sustainable development in aesthetic education, grounded in a comprehensive evaluation model.

In terms of practical application, the model proposed in this paper can assist policy makers and educational administrators in identifying the key points of investment in aesthetic education, optimising the funding allocation strategy, and ensuring the effective use of resources. Analysing the derivative curves enables educational administrators to dynamically evaluate the responsiveness of aesthetic education development to inputs and the effectiveness of those inputs. This provides data support for the continuous improvement of the effectiveness of the aesthetic education teaching and learning. By identifying the precursor points of rapid development, the model can facilitate colleges and government departments to formulate long-term planning and predict the future trend of aesthetic education development. The points at which both the first and second derivatives’ extreme values equal zero signify a pivotal shift in the sensitivity of funding inputs to the advancement of aesthetic education. These points corroborate the hypothesis that the field is poised for rapid development. In terms of model validation, in future studies, the authors will apply the model to the data on aesthetic education in other provinces to validate the generalisability of the model. In addition, the accuracy of the model predictions will be verified by tracking the actual data on the development of aesthetic education in Anhui Province over time. This paper constructs a comprehensive evaluation model of aesthetic education in universities in Anhui Province based on principal component analysis. Besides, it identifies the precursor points for the rapid development of aesthetic education, and defines the probability value of the sustainable development of aesthetic education in Anhui Province. The paper’s model excels by accommodating the discrete nature of data, making it versatile for comprehensive aesthetic education evaluations across diverse datasets. Yet, its efficacy is critically dependent on the quality and completeness of aesthetic education indicator data.

In addition, the model mainly analyses the relationship between the investment in aesthetic education and the development degree, failing to comprehensively consider other factors affecting the development of aesthetic education, such as cultural background, policy environment, and students’ interests. As artificial intelligence advances rapidly, the authors intend to leverage AI technology in their forthcoming research for mining and analysing aesthetic education big data. This will encompass a broader spectrum of data related to aesthetic education, including student feedback, assessments of teaching quality, and the social impact of these educational efforts. More comprehensive analytical support will be provided. Furthermore, the authors aim to employ deep learning models to assess the collective impact of various factors on the progression of aesthetic education. These models will predict how the field may evolve under diverse conditions, thereby enhancing the precision of decision-making support. On this basis, natural language processing technology will also be utilized to analyse literature, policy documents and expert opinions related to aesthetic education. Valuable information can be extracted for model optimisation and strategy formulation, while an intelligent recommendation system can be developed to recommend optimal aesthetic education development strategies and resource allocation plans according to the specific conditions of universities. In this case, the efficiency and effectiveness of aesthetic education work can be comprehensively enhanced. The paper’s research contribution is exemplified through the case study of Anhui Province, where it introduces a comprehensive evaluation model for aesthetic education. Besides, it defines the probability value of sustainable development of aesthetic education in Anhui Province. The paper’s model stands out for its ability to handle the discrete nature of data, making it suitable for conducting comprehensive evaluations of aesthetic education across a range of data scenarios. However, the size of the data volume may affect the analysis scope of the model, yet this limitation does not impact data accuracy or the model’s predictive trends and policy suggestions for aesthetic education. The authors plan to collect a larger volume of data in future studies to enhance the comprehensive evaluation model’s analysis capabilities.

## 7. Conclusion

Following a thorough evaluation of art education development across 46 universities in Anhui Province utilizing a construction of 25 indicators, it becomes evident that there are variations in the development of art education among these universities. For instance, Anhui University and Anhui Normal University, representing comprehensive and normal universities respectively, rank higher in terms of overall strength and university rankings. However, universities such as the University of Science and Technology of China, Hefei University of Technology, and Anhui Medical University, despite their high overall strength, do not necessarily lead in art education. Despite advancements in higher education, art education continues to be a weak aspect within the system. However, universities such as Chuzhou University and Huangshan University, while ranking average in overall university standings, demonstrate relatively advanced development in art education.

In summary, several observations arise: Firstly, universities housing art colleges boast advantages in faculty expertise. However, there is still a need to differentiate between specialized courses for art students and those for the general public. Secondly, universities without art colleges generally offer public arts courses in literature and culture. Thirdly, while some universities offer distinctive interdisciplinary courses such as Contemporary Science and Technology Arts (USTC), Medical Humanities Film (BBMC), High-tech Ceramic Materials (AHUT), and Architectural Aesthetics (AHJZU), there is still a relative scarcity of courses focusing on interdisciplinary studies and fostering students’ innovative thinking.

According to the undergraduate talent training regulations of the 46 universities, students must complete 2 credits of public arts courses to graduate during their school years. However, most universities have not fully offered public arts courses, leading to varying degrees of imbalance between supply and demand. Consequently, students may not necessarily fulfill their 2-credit art education based on their interests, hobbies, and specialties. Art education may even become utilitarian education driven by credit orientation, neglecting the importance of aesthetic consciousness, personality development, and innovative thinking, making it necessarily important to strengthen faculty construction and curriculum system development for public arts courses.

Among the forty-six universities in the province, the total number of student art groups and art-related clubs ranges from 5 to 30. However, a higher quantity of literary and art clubs does not necessarily indicate more artistic practice activities, as some clubs may be dormant. Indeed, art education extends beyond formal coursework in public arts courses; it encompasses practical experiences like visiting art galleries, museums, and attending art exhibitions. Additionally, various professional education programs also incorporate elements of art education. Therefore, the construction of a comprehensive evaluation index system for faculty strength, curriculum design, teaching management, artistic practice, and teaching support matters considerably for improving the quality of higher education. The next step in enhancing art education involves focusing on faculty quality, uniqueness, practicality, and innovation in the reform of public arts course teaching.

Herein, a comprehensive evaluation model for the development of art education in Anhui Province is constructed based on principal component analysis. This model quantifies the impact weights of various art education indicators on the development of art education in Anhui Province, and also provides a theoretical basis for identifying the precursors of rapid development of art education in Anhui Province. Furthermore, a new method for determining the development of art education in Anhui Province is also proposed, which takes into account the discreteness of art education indicator data in Anhui Province. The comprehensive evaluation model curve for art education development in Anhui Province exhibits an overall declining trend. Building upon this observation, the authors suggest utilizing the maximum value of the first derivative of the comprehensive evaluation model as the precursor point for rapid art education development in Anhui Province. This very approach leads to the definition of a probability equation for the sustainable development of art education in the province, forging a theoretical foundation for its future development.
